# Association between social support and anxiety among pregnant women in the third trimester during the coronavirus disease 2019 (COVID-19) epidemic in Qingdao, China: The mediating effect of risk perception

**DOI:** 10.1177/0020764020941567

**Published:** 2020-07-09

**Authors:** Chongyu Yue, Cuiping Liu, Jing Wang, Meng Zhang, Hongjing Wu, Chunrong Li, Xiuling Yang

**Affiliations:** 1 Department of Obstetrics, Affiliated Hospital of Qingdao University, Qingdao, China; 2 School of Nursing, Qingdao University, Qingdao, China; 3 Department of Neurosurgery, Affiliated Hospital of Qingdao University, Qingdao, China

**Keywords:** Social support, anxiety, risk perception, pregnant women, COVID-19

## Abstract

**Background:**

Coronavirus disease 2019 (COVID-19) is a public health emergency of international concern and poses a threat to the mental health of pregnant women.

**Aim:**

The purpose of this study was to investigate the relationship between social support and anxiety, and the mediating effect of risk perception during the COVID-19 epidemic in the third trimester pregnant women in Qingdao, China.

**Methods:**

From 16 to 21 February 2020, an online survey was conducted, which collected the information on demographic data, anxiety, social support and risk perception to COVID-19 of women with established medical records in the ambulatory of the Department of Obstetrics at the Affiliated Hospital of Qingdao University. Anxiety was assessed by the Self-Rating Anxiety Scale (SAS), social support was assessed by the Social Support Rating Scale (SSRS) and risk perception was assessed by a self-designed questionnaire.

**Results:**

This study had 308 participants with an average of 31.02 ± 3.91 years. During the period of prevention and control of the epidemic, most pregnant women adopted protective measures, such as wearing masks (97.4%), washing hands frequently (88.3%) and staying at home (76.3%). The average SAS, SSRS and risk perception scores of the participants were 42.45 ± 6.98, 44.60 ± 7.00 and 21.60 ± 5.74, respectively. The total effect of maternal social support on anxiety was −2.63 (95% confidence interval (CI): −4.40 ~ −1.44, *p* < .001), the direct effect was −1.44 (95% CI: −2.74 ~ −0.35, *p* < .05) and the indirect effect was −1.19 (95% CI: −2.49 ~ −0.51, *p* < .001).

**Conclusion:**

The third trimester pregnant women had a high level of social support, a medium level of risk perception to COVID-19 and were susceptible to anxiety. Risk perception played a mediating role between social support and anxiety.

## Introduction

Coronavirus disease 2019 (COVID-19) appeared in Wuhan, Hubei province in late December 2019, and then spread rapidly to other parts of China (Ahn et al., 2020). On 21 January 2020, the first confirmed case of COVID-19 was reported in Qingdao, where subsequently, the number of confirmed cases gradually increased. On 30 January 2020, confirmed cases were reported in all 31 provinces in China and in 18 other countries (Harapan et al., 2020). That same day, the World Health Organization (WHO) considered COVID-19 to be a public health emergency of international concern given the seriousness of the outbreak.

Infectious diseases epidemics not only damage the physical health of patients, but also have a tremendous psychological impact on the general public. The physical damage rooted in major public health events can be recovered in a short time, but the psychological consequences may persist for a much longer time. As a public health emergency, COVID-19 occurs suddenly, is highly contagious and lacks specific drugs. In the absence of timely treatment, the patient’s condition deteriorates rapidly and may even be fatal. The internet has been flooded with all kinds of information about the epidemic since the outbreak began. The epidemic information overload has caused an immense psychological effect on the general public, making them more likely to develop fear, anxiety or depression.

Pregnant women are generally more susceptible to respiratory pathogens, which include severe acute respiratory syndrome coronavirus (SARS-CoV), Middle East respiratory syndrome coronavirus (MERS-CoV) and COVID-19 (Qiao, 2020; Schwartz & Graham, 2020). Disease susceptibility may increase risk perception of the disease in pregnant women. Increased risk perception during pregnancy can lead to a number of consequences, including increased anxiety levels (Lennon, 2016). A previous study showed that anxiety is one of the most common negative emotions during pregnancy, being more frequent in the third trimester (Silva et al., 2017). However, it is uncertain how the risk perception of COVID-19 affects the level of maternal anxiety during the epidemic.

Social support refers to mutual material and spiritual support between individuals, as well as the exchange of material and spiritual resources between them, so that individuals can obtain the satisfaction of social needs (Zhang et al., 2020). Social support includes subjective and objective support, and its utilization. Previous studies have shown that a high level of social support plays a protective role in anxiety during pregnancy (Gümüşsoy et al., 2020; Nath et al., 2019). Social support is also one of the factors that influence risk perception and they are negatively correlated (Rui et al., 2009).

Thus, it is theorized that risk perception affects anxiety through two primary effects: a direct effect and a moderating effect. Previous studies consider that risk perception can directly affect anxiety among different populations (Roth et al., 2015; Takebayashi et al., 2017). Besides, some studies have supported that risk perception has mediating roles (Ban et al., 2019; Xu et al., 2016). A previous study demonstrated that risk perception can moderate the effects of social support on health behavior intention (Deng & Liu, 2017). Based on the evidence above, we hypothesized that risk perception may mediate the association between social support and anxiety in the third trimester pregnant women.

**Figure 1.**
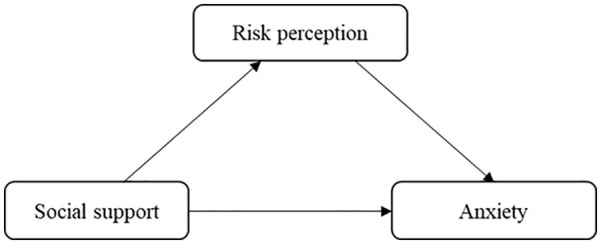
The conceptual model of the research.

Almost all current studies on COVID-19 are focused on epidemiological and clinical studies, and studies on maternal mental health are lacking. Therefore, the purpose of this study is to construct a structural equation model that explores the relationship between social support, risk perception and anxiety in the third trimester pregnant women. Thus, it seeks to reveal the correlation between social support and anxiety and whether this is mediated by risk perception. The hypothesized model is shown in Figure 1.

## Method

### Participants

Using the convenience sampling method, women in the third trimester of pregnancy with established medical records in the ambulatory of the Department of Obstetrics at the Affiliated Hospital of Qingdao University were selected as the research participants. This study was conducted using an online questionnaire in Chinese from 16 to 21 February 2020, within 1 month after confirmation of the first case of COVID-19 in Qingdao. The total number of late pregnant women in the ambulatory of the Department of Obstetrics at the Affiliated Hospital of Qingdao University was 750 from 16 to 21 February 2020. The formula for calculating the sample size is as follows (Charan & Biswas, 2013)

$$\begin{array}{l} n=\left(\;Z_{\alpha }^{2}\;\pi (1-\pi )\right)/\delta^{2}\\ \;=\left(1.96^{2}\times 0.25(1-0.25)\right)/0.05^{2}=288 \end{array}$$

Considering the non-response rate and inefficiency of the sample, the sample size was finally determined to be 380. This study was approved by the ethics committee of the Affiliated Hospital of Qingdao University.

### Measures

The self-report questionnaire had four parts: demographic characteristics, social support, risk perception and anxiety.

Demographic characteristics were collected, including age, education, gestational age, marital status and protective measures adopted.

A questionnaire was designed to evaluate how the third trimester pregnant women perceived the risk during the COVID-19 epidemic. The design of the questionnaire was based on the psychometric paradigm widely used in human health risk perception (Slovic, 1992). The 5-point Likert-type scoring method was used in the questionnaire. The total score was 1–40 and the higher the score, the higher the risk perception level. After compiling the questionnaire, a preliminary survey of 50 participants was conducted, and reliability and validity tests were performed. Cronbach’s α obtained in this study was .867, demonstrating good reliability in the data obtained.

Self-Rating Anxiety Scale (SAS) was compiled by Zung (1971) and was used to assess the subjective feelings of anxiety symptoms in pregnant women in this study. The SAS consists of 20 questions, graded on a scale of 1–4. Each question has four answer options: (1) *no or little time*, (2) *a small amount of time*, (3) *a considerable amount of time* and (4) *most or all of the time*. The score for the forward questions is 1, 2, 3 and 4 and the score for the reverse questions (5, 9, 13, 17 and 19) is 4, 3, 2 and 1. The standard score is obtained by multiplying the total score by 1.25. Participants who score less than 50 are free from anxiety, while those who score between 50 and 59 are mild anxious. Those who score 60–69 are moderate anxious and those who score greater than or equal to 70 are severe anxious. SAS has been widely used and has high reliability and validity (Minglu et al., 2020).

The Social Support Rating Scale (SSRS) was compiled by Shuiyuan Xiao (1994) and contains 10 questions divided into three dimensions: subjective support (questions 1, 3–5), objective support (questions 2, 6–7) and utilization of support (questions 8–10). Answers to questions 1–4 and 8–10 received 1–4 points. As for question 5, according to the support degree of the a–d options, each option is counted as 1–4 points. The answers to questions 6 and 7 received 0–9 points, depending on the source of support. The total score of the three dimensions is the total score of the scale. The total score ranges from 12 to 66. The higher the score, the higher the level of social support. Generally, the total score does not exceed 22, which indicates a low level of social support. A total score between 23 and 44 indicates a medium level of social support. Finally, a total score between 45 and 66 indicates a high level of social support. SSRS has been widely used and has high reliability and validity (Shi et al., 2020).

SPSS 19.0 software was used to detect the common method deviation by Harman’s single-factor test, according to the recommendations of Podsakoff et al. (2003). Descriptive analysis was used to calculate the scores of social support, risk perception and anxiety of pregnant women in the third trimester. Pearson’s correlation analysis was used to investigate the relationship between risk perception, social support and anxiety in these women. Amos 17.0 was used to establish a structural equation model between risk perception, social support and anxiety in pregnant women in the third trimester to test the mediating effect of risk perception on social support and anxiety in these women. In this study, *p* values < .05 were considered to be statistically significant.

The structural equation model was used to explore the relationship between risk perception, social support and anxiety in pregnant women in the third trimester. Social support was considered to be an endogenous latent variable and independent variable in the model. The three dimensions of objective support, subjective support and utilization of support were used as observed variables. Risk perception was considered as the observed variable that acts as a mediator in the model. Finally, anxiety was the observed variable and dependent variable in the model. The maximum likelihood method was used to fit the initial model. The fitting degree was tested by the ratio of chi-square to degree of freedom (χ^2^/*df*), root mean square error of approximation (RMSEA), comparative fit index (CFI), normed fit index (NFI), incremental fit index (IFI), Tucker–Lewis index (TLI) and goodness of fit index (GFI). Path coefficients were used to examine the action paths of social support on anxiety (including direct and indirect effects). The bootstrap method was used to analyze the mediating effect of risk perception.

## Results

### Test of common method deviation

Before the statistical analysis, common method deviation was analyzed by Harman’s single-factor test. The results showed that a total of 11 eigenvalues were greater than 1. In addition, the amount of variation explained by the first factor was 19.98%, which was well below the critical standard of 40% and it did not indicate obvious deviation from the common method. In this study, no single factor explained most of the variance found and the homologous error in the survey data was well controlled.

### Demographic characteristics

A total of 380 pregnant women in the third trimester were approached, of whom 58 women did not answer the questionnaire and 14 did not complete the questionnaire correctly, with a response rate of 81.1%. So, 308 pregnant women were included in the final analysis. As presented in Table 1, all the participants were married. Most of the participants had college and bachelor degrees (44.2%). The mean age was 31.02 ± 3.91 years, ranging from 21 to 42 years, and the mean gestational age was 31.63 ± 2.22 weeks, ranging from 28 to 36 weeks. During the period of prevention and control of the epidemic, most of the pregnant women adopted protective measures such as wearing masks (97.4%), washing their hands frequently (88.3%), staying at home (76.3%) and using household disinfectants for home disinfection (57%). Other protective measures, such as medical disinfectants (34.7%) and eye patches (11.7%), were used by a minority of pregnant women. Most of the pregnant women (84.7%) adopted three or more types of protective measures.

**Table 1.** The demographic and obstetric characteristics of the participants (*n* = 308).

	N/*M*	% (*SD*)
Age (years)	31.02	3.91
Gestational age (weeks)	31.63	2.22
Marital status		
Married	308	100
Unmarried/divorced	0	0
Education level		
High school or below	21	6.8
College degree	103	33.4
Bachelor’s degree	136	44.2
Master’s degree or above	48	15.6
Protective measures adopted		
Masks		
Yes	300	97.4
No	8	2.6
Eye patches		
Yes	36	11.7
No	272	88.3
Washing hands frequently		
Yes	272	88.3
No	36	11.7
Staying at home		
Yes	235	76.3
No	73	23.7
Household disinfectants		
Yes	176	57.1
No	132	42.9
Medical disinfectants		
Yes	107	34.7
No	201	65.3

### Anxiety and social support characteristics

The average SAS score of the participants was 42.45 ± 6.98, which is significantly higher than the Chinese norm of 37.23 ± 12.59 (*t* = 13.12, *p* < .05) (Yunyong et al., 2016) and that of late pregnant women (40.09 ± 7.40) prior to COVID-19 (*t* = 5.93, *p* < .05) (Ma et al., 2019), and is similar to that of the first line medical staff (42.79 ± 8.50) during the outbreak of COVID-19 (*t* = −0.85, *p* > .05) (Zhu et al., 2020). Of a total of 308 pregnant women, 14.3% (44 women) had an anxiety level above the standard score (SAS > 50), 1.6% (5 women) had a moderate anxiety level (SAS score between 60 and 70) and 0.3% (1 woman) had a high anxiety level (SAS score ⩾ 70).

The average SSRS score of the pregnant women was 44.60 ± 7.00, which is also significantly higher than the Chinese norm of 40.12 ± 5.11 (*t* = 8.72, *p* < .05) (Xiao, 1999). No pregnant woman had a total SSRS score below 22, which means a low level of social support. The average scores of subjective support, objective support and utilization of support were 25.35 ± 4.59, 10.06 ± 2.63 and 8.19 ± 1.93, respectively.

### Risk perception characteristics

In this study, the Kaiser–Meyer–Olkin (KMO) value was 0.86, and the χ^2^ value of Bartlett sphericity test was 1,129.96 (28 *df, p* = .000). These indices reached a highly significant level, indicating that there were common factors between the correlation matrices of the data groups and that it was suitable for factor analysis. Through variance maximization rotation principal component analysis, three factors were obtained, which explained a total variance of 73.59% (Table 2). Factor 1 included two questions related to the probability of COVID-19 infection, so it could be called ‘probability’. Factor 2 included two questions related to the COVID-19 severity risk, so it could be called ‘severity’. Factor 3 included four questions related to the concern raised by COVID-19, so it could be called ‘concern’. The average score of risk perception was 21.60 ± 5.74, indicating a medium level of risk perception. The average scores of probability, severity and concern were 5.00 ± 1.86, 4.12 ± 1.51 and 12.48 ± 3.33, respectively.

**Table 2.** Risk perception factor analysis.

Items	Loading
Probability	
I think that I may have COVID-19 during the prenatal testing.	0.73
I think that I may have COVID-19 even at home.	0.77
Severity	
I think I have little control over whether I would get infected or not.	0.75
I don’t think wearing a mask alone is a good way to protect against COVID-19.	0.80
Concern	
I feel extra pressure during the hospital visit.	0.84
I get nervous when I think about the threat of COVID-19.	0.83
I get nervous when I’m in close contact with a healthcare worker or other patients.	0.65
I think the hospital visit would put me at great risk, so I have a phobia about prenatal testing.	0.66

### Correlation analysis between the main variables

As shown in Table 3, the results of Pearson’s correlation analysis showed that risk perception was negatively correlated with social support (*r* = −.26, *p* < .01), while it was positively correlated with anxiety (*r* = .44, *p* < .01). Social support, in turn, was negatively correlated with anxiety (*r* = −.27, *p* < .01).

**Table 3.** The correlation coefficient between social support, risk perception and anxiety.

Variable	Social support	Risk perception	Anxiety
Social support	1.00		
Risk perception	−0.26**	1.00	
Anxiety	−0.27**	0.44**	1.00

** *p* < .01.

### Mediating effect of risk perception

**Figure 2.**
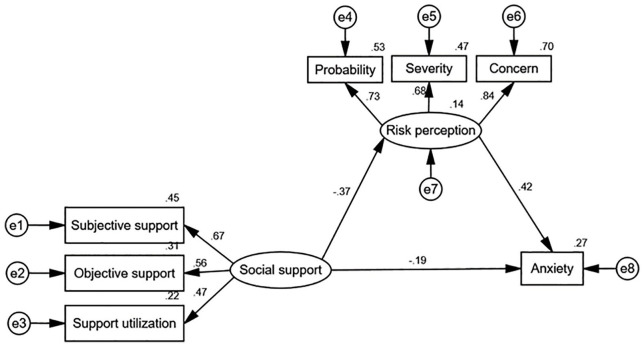
Mediating model of risk perception between social support and anxiety in pregnant women in the third trimester.

In this study, maternal anxiety was taken as the dependent variable, social support as the independent variable and risk perception as the mediating variable. A hypothesis model was established and the hypothesis relationship was tested using the structural equation model. The path diagram and the path coefficient between the variables were shown in Figure 2. The model fitting index showed that χ^2^/*df* was 1.228, RMSEA was 0.027, CFI was 0.994, NFI was 0.969, IFI was 0.994, TLI was 0.990 and GFI was 0.986. These indexes were all within the acceptable range, indicating that the model had a good fit. The path coefficients of social support and anxiety (β = −0.19, *p* < .05), social support and risk perception (β = −0.37, *p* < .01), and risk perception and anxiety (β = 0.42, *p* < .01) were all statistically significant.

Based on the multiple mediator testing process proposed by Preacher and Hayes (2008, this study performed 2,000 bootstrap tests on the mediating effect. The results showed that the path differences in the model were statistically significant. The total effect of maternal social support on anxiety was −2.63 (95% CI: −4.40 ~ −1.44, *p* < .001), the direct effect was −1.44 (95% CI: −2.74 ~ −0.35, *p* < .05) and the indirect effect was −1.19 (95% CI: −2.49 ~ −0.51, *p* < .001; Table 4). Therefore, risk perception had a mediating role between social support and anxiety, and the mediating contribution rate was 45.3%.

**Table 4.** Bootstrap test of the mediating effect of social support on anxiety.

Pathways	Estimate	Standard error	95% CI	*p*
Total effect				
Social support→anxiety	−2.63	0.79	−4.40 to −1.44	.001
Direct effect				
Social support→anxiety	−1.44	0.63	−2.74 to −0.35	.015
Social support→risk perception	−0.55	0.22	−1.06 to −0.25	.001
Risk perception→anxiety	2.15	0.42	1.36 to 3.05	.001
Indirect effect				
Social support→risk perception→anxiety	−1.19	0.53	−2.49 to −0.51	.001

CI: confidence interval.

## Discussion

This study was devised to explore the moderating effect of risk perception on the relationship between social support and anxiety symptoms. The results verified the assumption that risk perception moderates the relationship between social support and anxiety symptoms among women in the last trimester of pregnancy. According to the suggestion of Podsakoff et al. (2003), Harman’s single-factor test was applied in this study and the results revealed that there was no obvious common method deviation in the study. This testifies the reliability of the results presented here.

In this study, 308 pregnant women in the third trimester were investigated. The results showed that the incidence of anxiety in this group was 14.3%. The anxiety level of these women was higher than that of the general population prior to COVID-19, including the pregnant and non-pregnant population. The pregnant women’s anxiety level was higher than that of the healthcare workers in the hospital not receiving COVID-19 patients (Wang et al., 2020) and was comparable to that of the medical staff in the hospital receiving COVID-19 patients during the outbreak of COVID-19 (Zhu et al., 2020). Pregnant women, as a special group, are prone to develop anxiety and other adverse emotions. The rapid growth of the fetus makes the organs of the mother closer to the maximum functional load in the third trimester. Physical discomfort, fear of childbirth and concern for the fetus health lead to mental stress in pregnant women (Silva et al., 2017). At the same time, adrenocortical hormone secretion is increased in pregnant women, which makes them prone to anxiety and other adverse emotions. In addition, large-scale infectious diseases result in an inevitable increase in the public level of anxiety (Huang & Zhao, 2020). The incidence of maternal anxiety was higher during the epidemic in this study than during the non-epidemic period reported in Kirupamani’s study (Viswasam et al., 2019). The COVID-19 outbreak occurred suddenly and, given the current situation, is likely to continue for an extended period of time, which can cause psychological stress. The incidence of anxiety increased in several different groups during the COVID-19 epidemic (Cao et al., 2020; Chen et al., 2020; Choi et al., 2020; Huang & Zhao, 2020).

The pregnant women evaluated in this study had a higher level of social support than the general population, similar to a previous study (Gao et al., 2014). In China, pregnancy is a joy for the whole family, especially in the third trimester. The whole family will do everything possible to care for the late pregnant woman so that she does not suffer any accident near the time of delivery. In addition, the Chinese government has issued a series of laws, regulations and preferential policies for prenatal care and pregnant women, which protect the legitimate rights and interests of these women. At present, free schools for pregnant women in major hospitals are also an important source of formal social support during pregnancy. The role of these schools is mainly reflected in maintaining health during pregnancy and in disseminating healthcare knowledge (Wei et al., 2018). During the epidemic period, many hospitals, including the hospital where this survey was conducted, launched different online courses to answer questions and provide pregnant women with health guidance during pregnancy and protective measures for the epidemic situation. The average age of the pregnant women in this study was 31.02 ± 3.91 years, which is the average for the normal population of childbearing age. All woman in this study were outpatient patients with no common diseases during pregnancy. These factors increase the utilization of social support for pregnant women.

This study shows that social support was negatively correlated with anxiety. Social support has a direct negative influence on the anxiety of pregnant women, which is consistent with previous studies (Biaggi et al., 2016; Gao et al., 2019). Social support can play a direct protective role in individuals’ negative emotions, by helping with behavior and providing emotional support. In addition, social support can also improve the assessment and coping skills of individuals, reduce the perceived severity of stressful events and thus play an indirect protective role in mental health (Lakey & Orehek, 2011). As an important source of social support, the care and support of family members, especially spouses, can alleviate the adverse effects of stressful life events on pregnant women. Meanwhile, good social support can also provide a good individual emotional experience in non-stressful circumstances.

The risk perception of pregnant women at late trimester for COVID-19 assessed in this study was of medium level. Most of pregnant women adopted three or more types of protective measures (84.7%) and wore masks when leaving home (97.4%). The Chinese government has taken strong and effective measures to prevent and control the epidemic. The government publishes daily updates on the outbreak, including the number of infections and confirmed patients’ movements. These factors may be the reason why the risk perception of pregnant women was medium rather than high. However, even a moderate risk perception can increase anxiety levels in pregnant women and mediate the relationship between social support and anxiety. Bayrampour et al. (2013) showed that the higher the risk perception level of pregnant women, the more severe the anxiety level. Therefore, medical teams should make the risk perception level of pregnant women precise by spreading accurate information to them, to reduce their anxiety levels.

The results obtained here show that social support could regulate anxiety directly and negatively or affect it indirectly through risk perception. Social support was negatively correlated with risk perception, while risk perception was positively correlated with anxiety. Risk perception moderates the relationship between social support and anxiety negatively. Thus, during the epidemic, health professionals can take two measures to maintain the mental health of pregnant woman and reduce anxiety: actively mobilize the social support system for pregnant women and reduce the risk perception level of pregnant women in relation COVID-19.

The present study provides new insights into the mediating effect of risk perception of pregnant woman on social support and anxiety. However, it presents some limitations that should be addressed. A comparative study between pregnant women and the general population has not been performed due to the lack of data on anxiety level in the general population during the COVID-19 outbreak. In addition, the present study may contain a selection bias because only pregnant women who underwent prenatal examination at a single hospital outpatient clinic were selected to participate in the survey. Finally, as a self-report instrument was used to collect data from this study, pregnant women may have over- or underreported their data.

## Conclusion

The third trimester pregnant women evaluated here had a high level of social support, a medium level of risk perception to COVID-19 and constituted a vulnerable population with a high risk of developing anxiety. Maternal anxiety was strongly associated with social support and risk perception in this study. In addition, it was observed that social support can affect anxiety, directly or indirectly, through risk perception. Therefore, healthcare professionals should endeavor to strengthen the social support of pregnant woman and reduce their risk perception, thereby decreasing their anxiety.
